# Quantification and Classification of Contrast Enhanced Ultrasound Breast Cancer Data: A Preliminary Study

**DOI:** 10.3390/diagnostics12020425

**Published:** 2022-02-06

**Authors:** Georgios S. Ioannidis, Michalis Goumenakis, Ioannis Stefanis, Apostolos Karantanas, Kostas Marias

**Affiliations:** 1 Computational BioMedicine Laboratory (CBML), Foundation for Research and Technology—Hellas (FORTH), 70013 Heraklion, Greece; mgoumenakis@gmail.com (M.G.); jstefanis@ics.forth.gr (I.S.); karantanas@med.uoc.gr (A.K.); kmarias@ics.forth.gr (K.M.); 2 Department of Radiology, Medical School, University of Crete, 71003 Heraklion, Greece; 3 Department of Electrical & Computer Engineering, Hellenic Mediterranean University, 71410 Heraklion, Greece; 4 Department of Medical Imaging, University Hospital, 71003 Heraklion, Greece

**Keywords:** perfusion/models, breast carcinoma, contrast enhanced ultrasonography, prognostic factors, quantitative analysis

## Abstract

This study aimed to investigate which of the two frequently adopted perfusion models better describes the contrast enhanced ultrasound (CEUS) perfusion signal in order to produce meaningful imaging markers with the goal of developing a machine-learning model that can classify perfusion curves as benign or malignant in breast cancer data. Twenty-five patients with high suspicion of breast cancer were analyzed with exponentially modified Gaussian (EMG) and gamma variate functions (GVF). The adjusted R^2^ metric was the criterion for assessing model performance. Various classifiers were trained on the quantified perfusion curves in order to classify the curves as benign or malignant on a voxel basis. Sensitivity, specificity, geometric mean, and AUROC were the validation metrics. The best quantification model was EMG with an adjusted R^2^ of 0.60 ± 0.26 compared to 0.56 ± 0.25 for GVF. Logistic regression was the classifier with the highest performance (sensitivity, specificity, G_mean_, and AUROC = 89.2 ± 10.7, 70.0 ± 18.5, 77.1 ± 8.6, and 91.0 ± 6.6, respectively). This classification method obtained similar results that are consistent with the current literature. Breast cancer patients can benefit from early detection and characterization prior to biopsy.

## 1. Introduction

Breast cancer constitutes the most common neoplasm in women and accounts for 30% of all recently diagnosed cancer in women [1]. Early detection and treatment are the most significant factors for reducing mortality and improving quality of life [2]. Mammography is the modality of choice for screening, which has been proven to reduce mortality due to breast cancer [3,4]. However, it exhibits low sensitivity (30–48%) in dense breasts [3], resulting in misdiagnosis and a high rate of false negative cases [5]. In addition, mammography exhibits limited diagnostic sensitivity (65.2%) for small lesions (≤1.0 cm) compared to ultrasound (85.1%), regardless of breast density [6]. Consequently, the majority of cancer-free women pay a high price in terms of false positive results, especially in the United States [7].

On the contrary, contrast enhanced ultrasound (CEUS) has been found to improve diagnostic efficacy compared to mammography or unenhanced ultrasound [8,9,10] because it also evaluates blood perfusion from tumor-induced neovascularity [11]. Published studies have shown that CEUS increases accuracy in distinguishing benign from malignant breast lesions having both qualitative [12] and quantitative [13] features. Furthermore, CEUS can improve the diagnosis of breast cancer in early stages because it can accurately differentiate benign from malignant lesions [14], and it can help to prevent unnecessary biopsies because of its high negative predictive value (100% on BIRADS III and 89.2% on BIRADS IV) [15]. Moreover, a recent systematic review of 51 studies with 4875 patients reported an high overall sensitivity reaching 88%. 

Aside from CEUS and mammography, dynamic contrast-enhanced MRI (DCE-MRI) is a popular and important tool for breast lesion characterization [16]. The large number of freely accessible breast cancer datasets led to an increase in studies on breast lesion classifications [17,18,19,20,21,22], which exhibit high accuracy and AUROC (above 80%) [23,24,25]. Additionally, in a recent radiomic analysis including contrast enhanced mammography data, the authors presented a high AUROC varying from 89% to 96% for classifying benign and malignant breast lesions [26]. By conducting bibliography research on computed tomography (CT), it is evident that no efforts have been devoted to breast lesion classification but only to breast tissue classification (i.e., fatty, glandular, or dense) [27].

CEUS is a perfusion imaging technique that uses perflubutane as a contrast agent. Vascular perfusion can be visualized in real time through the contrast effect of 2–3 μm perflubutane microbubbles [28,29]. Therefore, the dynamic scan can produce parametric maps of hemodynamic parameters via pharmacokinetic modeling. Hemodynamic parameters or parametric maps are limited because of the high cost of the commercially available products and the lack of freely accessible quantification software. Therefore, the majority of studies aiming to classify breast lesions as benign or malignant utilized qualitative or semantic tumor characteristics such as the shape, diameter, and boundaries, as presented in a recent systematic review [11].

The aim of this study was two-fold. Firstly, we investigated which semi-quantitative perfusion model better described the behavior of CEUS curves. Secondly, we built a machine-learning framework that used quantitative and statistical characteristics of each CEUS curve to classify breast lesions as benign or malignant. To the best of our knowledge, we are the first to develop a classification model directly from perfusion curves with the aim of characterizing each image voxel as benign or malignant. 

## 2. Materials and Methods

### 2.1. Study Population

From June 2019 to May 2021, we performed CEUS on a total of 27 breast lesions (BI-RADS IV) in 27 women at our local hospital. However, 2 patients were excluded due to patient motion during dynamic contrast enhanced ultrasound examination. Thus, 25 patients were included in the study. After each patient underwent ultrasound-guided biopsy, we determined that 14 had histopathologically proven breast cancer and the rest had benign lesions, mainly fibroadenomas. Patient characteristics can be found in Table 1.

The inclusion criteria were women who underwent conventional US for (a) a screening procedure or (b) the characterization of a known palpable lesion or (c) a detectable finding from the US that required biopsy. The exclusion criteria were women with (a) known metastatic breast cancer diagnosed by biopsy or (b) US findings that had not been confirmed by biopsy.

### 2.2. Imaging Protocol 

This study was conducted at the US department of “Venizeleio” General Hospital with an iU22 Ultrasound System (Philips, Bothell, WA, USA). A CEUS study was performed involving women with a suspicious ultrasound breast finding, for which a biopsy was required. The sample was sent for pathological examination, and the result were compared to the contrast enhancement pattern of the lesion. The pathology report served as the ground truth for the development of the CEUS classification model.

### 2.3. Data Pre-Processing 

**Figure 1:**
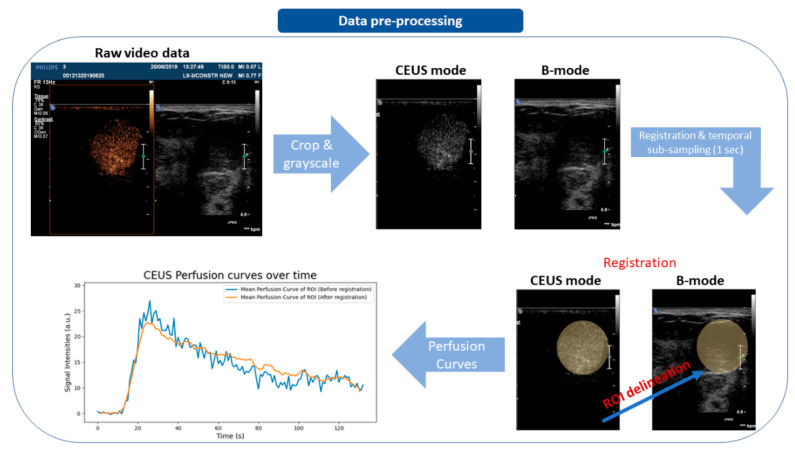
Data pre-processing workflow.

As raw DICOM CEUS data are usually stored in a multi-channel video format, the first step was to convert videos into grayscale using the luminance algorithm [30]. The next step was to extract B-mode and CEUS sequences with automated video-cropping techniques using the DICOM tag “SequenceOfUltrasoundRegions”. Subsequently, videos were temporally sub-sampled to 1 s resolution to facilitate the quantitative model-fitting process. In order to avoid motion artifacts, videos were registered in the temporal domain using the pyStackReg library https://pypi.org/project/pystackreg (accessed date: 10 October 2021) for Python [31]. Lastly, a clinical expert delineated the suspicious regions from which dynamic signal curves were extracted. The pre-processing steps are summarized in Figure 1.

### 2.4. CEUS Quantification, Parametric Mapping

In order to extract quantitative voxel-by-voxel markers from the perfusion curves, two functions were used to fit each curve. The first was the exponentially modified Gaussian (EMG) function, presented in Equation (1), and the second was the Gamma variate function (GVF), presented in Equation (2):

(1) $$F\left(t\right)=\frac{ac\sqrt{2\pi }}{2d}\exp\left(\frac{b-t}{d}+\frac{c^{2}}{2d^{2}}\right)\left[\frac{d}{\left|d\right|}-erf\left(\frac{b-t}{\sqrt{2c}}+\frac{c}{\sqrt{2d}}\right)\right]$$

where erf(t) is the Gaussian error function, $erf\left(t\right)=\int_{-t}^{t}e^{-x^{2}}dx.$

(2) $$G\left(t\right)={\;A\;t}^{a}\;\exp\left(-\frac{t}{b}\right)$$

In both equations, the unknown parameters a, b, c, and d for Equation (1) and A, a, and b for Equation (2) do not have physiological meaning; thus, optimization was performed in the range of real numbers using the Levenberg–Marquardt algorithm [32]. 

After fitting Equations (1) and (2) to the perfusion curves per voxel, a variety of semi-quantitative parameters could be computed using the first derivative of each of the functions such as: wash-in, wash-out, time to peak (TTP), and time to maximal slope (TMSP). More precisely, wash-in and wash-out describe the rate of change of contrast’s agent inflow and outflow, respectively. Mathematically, this is described as the maximum and minimum value of the derivative, respectively. TTP is the time required for Equations (1) and (2) to reach their maximum value. TMSP reflects the time point of maximum wash-in. Computationally, TMSP is the time required for the first derivative of Equation (1) to reach its maximum value. These equations have been used to describe the dispersion of a bolus as it passes through a series of compartments, mainly for perfusion modeling [33,34,35,36]. Further information and graphical representation of the quantitative markers for EMG can be found in [37,38].

Aside from the aforementioned imaging markers (wash-in, wash-out, TTP, and TMSP), we calculated the area under the perfusion curve (AUC) and the mean slope of increase (MSI). Assuming $C_{t}\left(t\right)$ to be the perfusion curve and $t_{0}$ to be the final time of the baseline, MSI was computed by the following formula:

(3) $$MSI=\frac{1}{N}\overset{t_{N}=TTP}{\underset{t_{1}=t_{0}}{\sum }}C_{t}\left(t_{i+1}\right)-C_{t}\left(t_{i}\right)$$

### 2.5. Goodness of Fit

The criterion chosen to assess the goodness of fit between model function and data (CEUS curves) was the adjusted R^2^ (${\bar{R}}^{2}$), a generalized metric that is based on R-squared $\left(R^{2}\right)$. This metric is suitable for the purpose of this study since it accounts for both the number of temporal points of the curve (N) and the number of explanatory variables (p) of the model [39]. ${\bar{R}}^{2}$ is given in Equation (4) and its values range from 0 to 1.

(4) $${\bar{R}}^{2}=1-\left(1-R^{2}\right)\frac{N-1}{N-p-1}$$

### 2.6. Machine-Learning Pipeline

#### 2.6.1. Feature Extraction

Since our dataset was limited in the number of enrolled patients, classification analysis was performed on a voxel-by-voxel basis using the perfusion curves from each ROI delineated by the expert. For the differentiation between benign and malignant tissue types from each ROI, two sets of features depending on the fitting equation were extracted. The first feature set (EMG set) included both statistical features and semi-quantitative metrics stemming from the EMG-fitted curve, as previously described. Thus, the first set of features (EMG set) included wash-in, wash-out, TTP, TMSP, AUC, MSI, mean, median, max, and standard deviation(SD). The GVF set was the same as the first and differed only in that the first 6 features were computed from the fitted GVF function on CEUS curves.

#### 2.6.2. Feature Selection

In order to make our model more robust and reliable, the minimum redundancy maximum relevance feature-selection algorithm from the pymrmr library [40] was used to identify the most relevant patterns in the training set. 

Minimum redundancy maximum relevance (MRMR) is a supervised feature-selection algorithm (i.e., uses both the input features and output class labels). The aim of MRMR is to find the set of features that best matches the output class labels while minimizing redundancy among selected features. In order to find the best match between the features and the output labels, MRMR usually deploys the mutual-information framework. Further information can be found in [40].

#### 2.6.3. Classification

Differentiation between benign and malignant breast lesions was achieved using a variety of classifiers obtained from the scikit-learn library [41] such as: quadratic discriminant analysis (QDA), Gaussian naïve Bayes (GaussianNB), AdaBoost, random forest, k-nearest neighbors (KNeighbors; k = 3), and logistic regression. We used a support vector machine (SVM) with the radial basis function kernel (RBF).

In the context of perfusion curves differentiation (benign or malignant), all classifiers were trained in a 10-fold cross-validation scheme on the extracted curve features. Data stratification was applied on a patient basis across folds, avoiding sample selection bias and overfitting of models.

#### 2.6.4. Model Evaluation Metrics

In order to evaluate the classification performance, the standard deviations of several metrics (for each fold) were calculated on the unseen testing sets. The metrics assessing performance included $sensitivity=\frac{TP}{TP+FN}$, $specificity=\frac{TN}{FP+TN}$, and geometric mean $G_{mean}=\sqrt{sensitivity\times specificity}$. TP, TN, FP, and FN stand for true positive, true negative, false positive, and false negative, respectively. The geometric mean aggregates both sensitivity and specificity, and it is suitable for imbalanced datasets. Moreover, the area under the receiver operating characteristic (ROC) curve (AUROC) was calculated. The ROC curve is a two-dimensional graph in which the y-axis indicates the true-positive rate and the x-axis indicates the false-positive rate. It has been extensively used to evaluate medical decision-making and machine-learning systems. Please note that AUROC differs from AUC, which is the area under the perfusion curve. 

## 3. Results

### 3.1. Goodness of Fit

The mean value ± the standard deviation of the goodness of fit metric ${\bar{R}}^{2}$ calculated from all voxels inside the suspicious areas for the two models, EMG and GVF, was 0.60 ± 0.26 and 0.56 ± 0.25, respectively.

**Figure 2:**
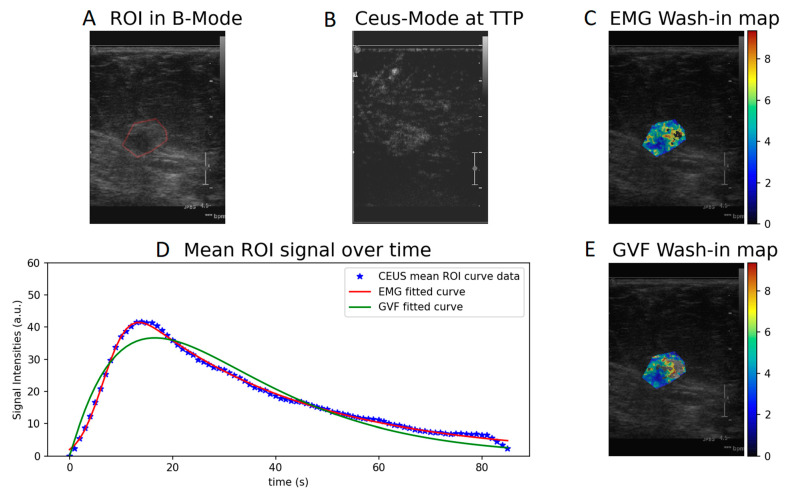
Wash-in parametric map calculated with EMG (**C**) and GVF (**E**) of a cancer patient. (**A**) ROI in B-mode, (**B**) CEUS mode at time to peak, and (**D**) EMG and GVF fitted to the mean ROI signal over time.

**Figure 3:**
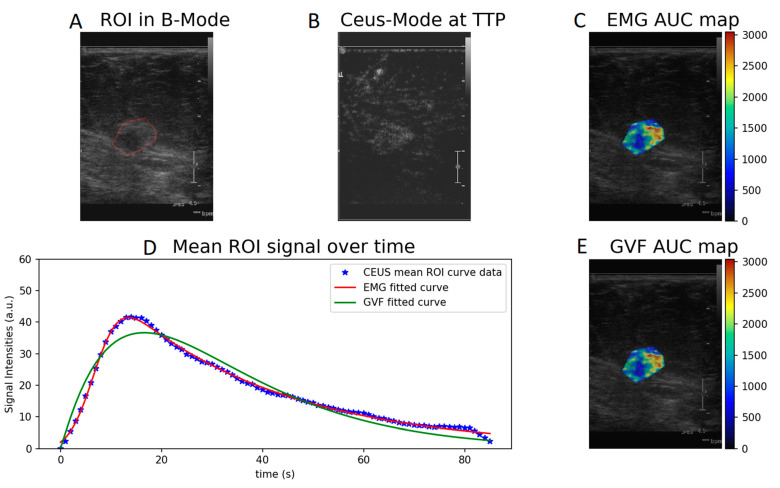
AUC parametric map calculated with EMG (**C**) and GVF (**E**) of a cancer patient. (**A**) ROI in B-mode, (**B**) CEUS mode at time to peak, and (**D**) EMG and GVF fitted to the mean ROI signal over time.

**Figure 4:**
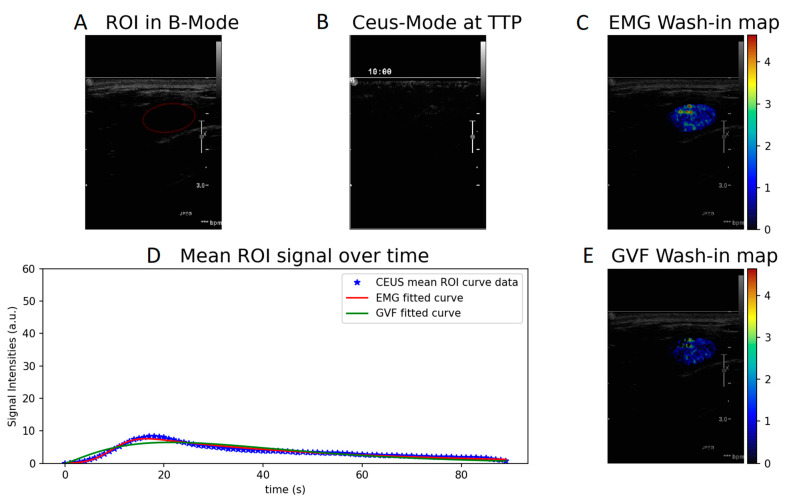
Wash-in parametric map calculated with EMG (**C**) and GVF (**E**) of a benign case. (**A**) ROI in B-mode, (**B**) CEUS mode at time to peak, and (**D**) EMG and GVF fitted to the mean ROI signal over time.

**Figure 5:**
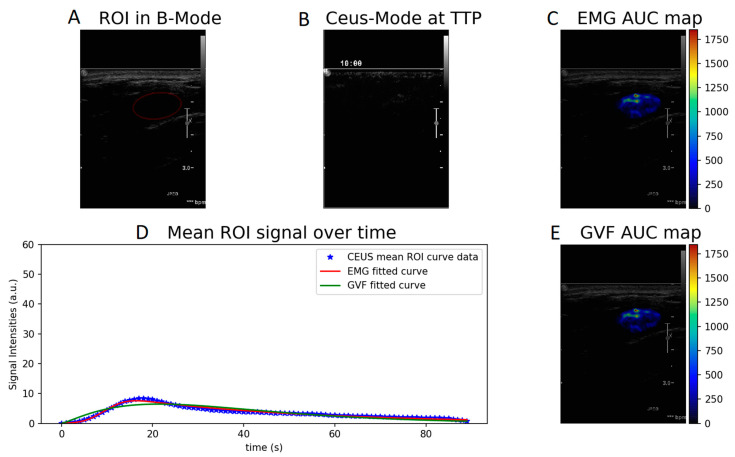
AUC parametric map calculated with EMG (**C**) and GVF (**E**) of a benign case. (**A**) ROI in B-mode, (**B**) CEUS mode at time to peak, and (**D**) EMG and GVF fitted to the mean ROI signal over time.

Wash-in- and AUC-produced parametric maps after voxel-by-voxel fitting to the CEUS perfusion data using Equations (1) and (2) are presented in Figure 2 and Figure 3. The parametric maps of a benign fibroadenoma patient are shown in Figure 4 and Figure 5. 

### 3.2. Machine Learning 

#### 3.2.1. Feature Selection

The best selected features from the EMG feature set were wash-in, AUC, SD, max, TTP, and mean. In addition, the best selected features from the GVF set were WIN, AUC, SD, Max, mean and wash-out.

#### 3.2.2. Classification Results

The metrics for the classification for benign and malignant tissue types are summarized in Table 2 for the EMG feature set and in Table 3 for the GVF feature set. 

## 4. Discussion

In this work, two functions were used to quantify CEUS perfusion signals into meaningful imaging markers with the goal of building a machine-learning model that can classify the perfusion curves as benign or malignant. 

Concerning the quantification of the CEUS curves, the model that better described CEUS perfusion was found to be the exponentially modified Gaussian function (EMG) according to the adjusted R^2^ criterion. This is a logical outcome since the EMG function has more degrees of freedom (four parameters) in fitting the CEUS data than the gamma variate function (three parameters). In addition, GVF model was found to be more sensitive to noise compared to EMG as it failed to quantify a non-negligible number of voxels. This could be attributed either to high level of noise in the temporal data or to numerical errors in the computation of the derivative, which is prerequisite for obtaining the wash-in parameter. This can be easily observed in the wash-in values (Figure 2C,E and Figure 4C,E) where several voxels appear without color. 

Quantitative models aim to describe physiology and use appropriate simplifications such as the number of compartments to derive a mathematical model. On the contrary, semi-quantitative methods are data-driven and do not attempt to model the underlying physiology. The major advantage of using semi-quantitative models for breast perfusion evaluation is the exclusion of the arterial input function (AIF). In addition, semi-quantitative models are less prone to numerical errors, having a one-step process as opposed to a more complex workflow [38]. In such acquisitions where the imaging field of view is focused on the lesion, and the area of the artery is frequently non-visible. This is a different approach from the widely used perfusion models such as the extended Tofts model, Patlak’s model, etc. [42,43], where the AIF is mandatory for quantification. 

Regarding the classification analysis, the EMG feature set (Table 2) expectedly performed better than the GVF feature set (Table 3) according to the AUROC metric. This metric was preferred over the others because it is indicative of the separability between classes because it takes into account sensitivity and specificity. Keeping that in mind, the logistic regression classifier exhibited the best performance with both feature sets. The geometric mean was incorporated in our study since it is a more suitable metric for handling unbalanced datasets [44,45]. Our machine-learning method is based on training classifiers with features obtained from every voxel inside the breast lesion, with 22,446 benign and 65,762 malignant voxels. 

CEUS has the potential to improve the diagnostic efficacy of mammography. Notably, the results of our study on breast cancer classification are in line with a recent review of 51 CEUS-based studies that reported a mean sensitivity of 0.88. and a mean AUROC of 0.91 [11]. 

To the best of our knowledge, this is the first study to distinguish benign and malignant tissue types using CEUS perfusion curves on a voxel-by-voxel basis. A similar approach was investigated by Ta et al., who reported an ACC of 100% in a cohort of 10 rat tumors [46]. As a result, our study lacks a point of comparison. There are similar studies, such as those on region-based quantitative classification analysis; Kapetas et al. reported an AUROC of 0.812% in a cohort of 65 patients [47], Janu et al. reported an AUROC of 78% in a cohort of 230 patients [48], and Park et al. reported an AUROC of 0.841 in a cohort of 98 patients [49].

Although this study exhibited promising results, there is room for improvement. For example, a larger patient cohort would make our analysis more statistically robust. In addition to the dynamic data that were used, a larger patient cohort would introduce more spatial and textural information to the model such as radiomics. 

## 5. Conclusions

In conclusion, we presented a novel approach based on dynamic CEUS signal curves for the classification of benign and malignant breast lesions. The EMG feature set exhibited the highest performance (AUROC 91%) in distinguishing malignant and benign lesions on a voxel-by-voxel basis. This framework has the potential to evolve into an objective diagnostic support tool using the dynamic signal characteristic of CEUS, reducing unnecessary biopsies in breast cancer screening programs.

## Figures and Tables

**Table 1:** Patient characteristics.

Characteristics	n
Total patients	25
Women	25
Age (in years)	
Mean	52.3
Median	50
Range	28–79
Histopathological grades	
BIRADS IV	25
Benign lesions	14
Malignant lesions	11
Number of benign voxels	22,446
Number of malignant voxels	65,762

**Table 2:** Classification metrics ± standard deviation per classifier using EMG feature set.

Classifiers	Sensitivity	Specificity	G_mean_	AUROC
QDA	69.7 ± 20.8	88.5 ± 12.0	76.8 ± 10.9	89.7 ± 5.4
GaussianNB	69.0 ± 22.1	90.7 ± 11.2	77.2 ± 12.5	89.8 ± 7.4
AdaBoost	87.4 ± 11.9	62.6 ± 21.5	72.2 ± 11.4	87.9 ± 9.7
Random forest	88.3 ± 11.8	70.3 ± 17.5	77.6 ± 9.2	87.1 ± 9.8
KNeighbors	85.4 ± 11.5	55.6 ± 15.1	67.9 ± 9.1	76.7 ± 9.2
Logistic regression	89.2 ± 10.7	70.0 ± 18.5	77.1 ± 8.6	91.0 ± 6.6
SVM	88.1 ± 11.4	68.6 ± 18.6	76.7 ± 11.1	87.9 ± 10.8

**Table 3:** Classification metrics ± standard deviation per classifier using GVF feature set.

Classifiers	Sensitivity	Specificity	G_mean_	AUROC
QDA	70.4 ± 21.5	83.8 ± 18.6	74.2 ± 12.5	87.6 ± 7.1
GaussianNB	67.8 ± 23.0	87.6 ± 19.3	74.2 ± 14.4	88.8 ± 6.6
AdaBoost	89.2 ± 11.3	57.1 ± 20.1	69.5 ± 10.6	86.6 ± 9.8
Random forest	90.4 ± 10.1	60.8 ± 25.8	71.9 ± 14.3	86.5 ± 11.8
KNeighbors	86.6 ± 9.8	52.9 ± 15.8	66.3 ± 8.7	76.1 ± 7.5
Logistic regression	88.5 ± 13.3	66.3 ± 22.4	74.6 ± 11.8	89.0 ± 11.0
SVM	89.2 ± 10.3	56.2 ± 25.1	68.1 ± 15.9	85.8 ± 9.2

## Data Availability

The data are available upon a reasonable request to the corresponding author.
